# Sociodemographic and clinical characteristics of suspected difficult-to-treat depression

**DOI:** 10.3389/fpsyt.2024.1371242

**Published:** 2024-08-21

**Authors:** Masami Murao, Yasuyuki Matsumoto, Mariko Kurihara, Yuki Oe, Izumi Nagashima, Tomonari Hayasaka, Takashi Tsuboi, Koichiro Watanabe, Hitoshi Sakurai

**Affiliations:** ^1^ Department of Neuropsychiatry, Kyorin University Faculty of Medicine, Tokyo, Japan; ^2^ Department of Occupational Therapy, Kyorin University Faculty of Health Sciences, Tokyo, Japan

**Keywords:** bipolar depression, diagnosis, difficult-to-treat depression, major depressive disorder, subthreshold depression

## Abstract

**Introduction:**

Difficult-to-treat depression (DTD) represents a broad spectrum of patients with persistent depression where standard treatment modalities are insufficient, yet specific characteristics of this group remain insufficiently understood. This investigation aims to delineate the sociodemographic and clinical profiles of suspected DTD patients in real-world clinical settings.

**Method:**

We conducted a retrospective analysis of data from patients comprehensively evaluated for suspected DTD at Kyorin University Hospital, Tokyo, Japan, between October 2014 and September 2018. The study participants consisted of individuals with persistent depression unresponsive to conventional antidepressant treatments during the current episode. Diagnoses adhered to the criteria of the Diagnostic and Statistical Manual of Mental Disorders, Fourth Edition, Text Revision. Additional evaluations included the Montgomery-Åsberg Depression Rating Scale (MADRS) and other pertinent measures. The analysis focused on comparing demographic and clinical characteristics across diagnosed groups.

**Results:**

The analysis encompassed 122 patients, with diagnoses of major depressive disorder (MDD) in 41.8%, bipolar disorder (BD) in 28.7%, and subthreshold depression in 29.5%. Notably, high incidences of psychiatric comorbidities were present across all groups, with anxiety disorders exceeding 30% and personality disorders surpassing 50%. The only significant distinction among the three groups was observed in the MADRS scores, with the MDD group exhibiting the highest values (20.9 ± 9.7 vs. 18.6 ± 9.3 vs. 11.3 ± 7.4, p<0.01).

**Conclusions:**

This study sheds light on the intricate nature of suspected DTD, emphasizing the coexistence of MDD, BD, and subthreshold depression within this category. Our findings underscore the necessity for thorough evaluations and tailored treatment approaches for managing suspected DTD.

## 1 Introduction

Depression is a significant mental health concern, affecting approximately 4.4% of the global population (1). Although around 89% of drug-naïve patients achieve remission within one year following their initial depressive episode with consistent antidepressant treatment, 40-70% of these individuals experience a relapse or recurrence within the next year (2). Acknowledging the necessity for specialized attention for patients who require more than the standard treatment approach, difficult-to-treat depression (DTD) has been recently conceptualized (3, 4). DTD refers to a wide range of depressive states that continues to impose a significant burden despite conventional treatment (5). In other words, DTD is characterized by suboptimal outcomes due to treatment failure, which includes non-response, intolerance, lack of acceptance, and contraindications across any treatment modality.

Since DTD encompasses a broad spectrum of clinical presentations, the diagnosed patients may exhibit a diverse array of clinical factors influencing their treatment response. To effectively manage patients suspected of having DTD, it is essential to assess a range of factors to ensure an accurate diagnosis. This consideration encompasses not only major depressive disorder (MDD) but also bipolar disorder (BD) and depression secondary to other psychiatric or neurological conditions (4). Subthreshold depression is also considered within the scope of suspected DTD due to their chronic nature, impact on daily functioning, and influence on long-term prognosis (6). Given that antidepressants typically show greater efficacy in MDD compared to other types of mood disorders, the treatment response in suspected DTD is likely to be influenced by the underlying diagnosis (7). Additionally, various patient-related factors including age, family history of mental illness, and personality characteristics, as well as illness-related factors like the duration and severity of the condition, coexisting anxiety disorders, developmental disorders, and intellectual level, also play a pivotal role (8–13). Therefore, a comprehensive diagnostic evaluation and clinical assessment are critical for predicting treatment outcomes in suspected DTD, allowing for a more tailored and potentially effective therapeutic approach.

To the best of our knowledge, however, there have been few previous studies that investigate the demographic and clinical characteristics of patients with suspected DTD in real-world settings. We therefore conducted cross-sectional research to examine the sociodemographic and clinical characteristics in patients who experienced persistent depression with significant burden despite several failed attempts at antidepressant treatment.

## 2 Methods

### 2.1 Design and participants

This study is a retrospective analysis of data from patients who underwent detailed assessment for suspected DTD at Kyorin University Hospital in Tokyo, Japan, from October 2014 to September 2018. The analysis included patients who (1) reported persistent depression, including secondary depression attributable to other psychiatric disorders, (2) experienced a substantial burden due to depressive symptoms leading to significant impairments in social functioning, (3) had failed antidepressant treatment during the current depressive episode, and (4) underwent a comprehensive assessment at Kyorin University Hospital. The institutional review board at Kyorin University approved the study (644-03). Due to the retrospective, non-interventional nature of the study and the use of pre-existing, de-identified data, obtaining informed consent from participants was deemed unnecessary. An opt-out option was available, enabling individuals to request the exclusion of their data from the analysis if desired.

### 2.2 Outcome measure

Medical, family, and social histories were meticulously reviewed via interviews with patients and their family members. Diagnoses were established in accordance with the Diagnostic and Statistical Manual of Mental Disorders, Fourth Edition, Text Revision (DSM-IV-TR), utilizing the Structured Clinical Interview for DSM-IV Axis I Disorders (SCID-I) and the Structured Clinical Interview for DSM-IV Axis II Personality Disorders. Additional assessments included the Montgomery-Åsberg Depression Rating Scale (MADRS) to evaluate depressive symptoms, the Young Mania Rating Scale (YMRS) for manic symptoms, the Autism-Spectrum Quotient (AQ) for autism-spectrum traits, the Adult Attention Deficit Hyperactivity Disorder Self-Report Scale (ASRS) for symptoms of attention deficit hyperactivity disorder (ADHD), and the Wechsler Adult Intelligence Scale-Third Edition (WAIS-III) to assess intelligence.

### 2.3 Statistical analysis

The patient records included data on age, sex, race, family history, diagnosis, duration of illness, university education, marital status, employment status, and the number of antidepressant treatment failure. Patients were categorized into three groups based on their diagnosis according to the SCID-I: those diagnosed with MDD (MDD group), BD (BD group), and those who have never experienced a major depressive episode (subthreshold depression group). Demographic and clinical characteristics across the three groups were compared using the Kruskal-Wallis test or the Fisher’s exact test. *Post hoc* multiple comparisons were performed using the Bonferroni correction following the Kruskal-Wallis test. A two-tailed P value of less than 0.05 was deemed statistically significant for all tests. All statistical analyses were performed using the Statistical Package for the Social Sciences software, version 27.0.1 for Windows (IBM Corporation, Armonk, NY).

## 3 Results

### 3.1 Patient diagnoses

Out of the 122 patients who reported persistent depressive symptoms, 51 patients (41.8%) were diagnosed with MDD, 35 (28.7%) with BD, and 36 (29.5%) with subthreshold depression. Within the BD group, 15 patients were classified into BD type I and 20 into BD type II. Two of the 36 patients with subthreshold depression did not receive any psychiatric diagnosis. Regarding the clinical background of the 122 patients, the mean age was 42.1 years, with 62 patients (50.8%) being female, 38 patients (31.2%) had a family history of mental illness, 11 patients (9.0%) had physical comorbidities, and 57 patients (46.7%) were married.

### 3.2 Sociodemographic characteristics

There were no significant differences in age, rate of females, physical comorbidities, education history, marital status, or social status among the three groups. There was a trend towards statistical significance in family history of mental illness (35.3% in MDD group, 40.0% in BD group, and 16.7% in subthreshold depression group, p=0.07) among the three groups **Table 1**.

**Table 1:** Sociodemographics of patients.

	MDD (n=51)	BD (n=35)	Subthreshold depression (n=36)	P-value
Age, years, mean (SD)	40.4 (12.1)	40.9 (11.2)	44.7 (14.0)	0.49
Female, n (%)	28 (54.9%)	19 (54.3%)	15 (41.7%)	0.42
Family history of mental illness, n (%)	18 (35.3%)	14 (40.0%)	6 (16.7%)	0.07
Physical comorbidities, n (%)	1 (2.0%)	5 (14.3%)	5 (13.9%)	0.12
University degree, n (%)	29 (56.9%)	19 (54.3%)	24 (66.7%)	0.52
Married, n (%)	20 (39.2%)	16 (45.7%)	18 (50.0%)	0.35
Social status, n (%)				0.22
Employed	6 (11.8%)	3 (8.6%)	4 (11.1%)	
Leave	12 (23.5%)	11 (31.4%)	10 (27.8%)	
Unemployed	21 (41.2%)	14 (40.0%)	14 (38.9%)	
Homemaker	7 (13.7%)	5 (14.3%)	6 (16.7%)	
Student	4 (7.8%)	2 (5.7%)	2 (5.6%)	

BD, bipolar disorder; MDD, major depressive disorder; SD, standard deviation.

### 3.3 Clinical characteristics

High incidences of psychiatric comorbidities were observed across the three groups. Notably, a substantial proportion of individuals in each group had personality disorders, with 76.5% in the MDD group, 77.1% in the BD group, and 50.0% in the subthreshold depression group (p=0.01). Among these, obsessive-compulsive personality disorder was present in 43.1% of the MDD group, 54.3% of the BD group, and 13.9% of the subthreshold depression group (p<0.01), while avoidant personality disorder was found in 33.3% of the MDD group, 37.1% of the BD group, and 8.3% of the subthreshold depression group (p<0.01). Other significant comorbid conditions included somatoform disorders (2.0% in MDD group, 2.9% in BD group, and 16.7% in subthreshold depression group, p<0.01) and alcohol use disorders (3.9% in MDD group, 14.3% in BD group, and 2.8% in subthreshold depression group, p<0.01). Anxiety disorders were also prevalent, affecting over 30% of individuals in each group (33.3% in MDD group, 34.3% in BD group, and 44.4% in subthreshold depression group, p=0.53).

Significant differences were observed in the MADRS total scores among the groups, with the MDD group scoring 20.5 ± 9.7, the BD group 18.6 ± 9.3, and the subthreshold depression group 11.3 ± 7.4 (p<0.01). Furthermore, when comparing each group, a significant difference was found only between the MDD group and the subthreshold depression group (p<0.01). The YMRS total scores also showed trend-level significance, with scores of 1.7 ± 3.0 in the MDD group, 4.4 ± 6.3 in the BD group, and 2.2 ± 2.9 in the subthreshold depression group (p=0.08). However, there were no significant differences in the duration of illness, the WAIS-III, the ASRS, and the AQ scores among the three groups. Additionally, the number of failed antidepressant treatments was as follows: 1.2 ± 0.9 for the MDD group, 0.9 ± 0.9 for the BD group, and 1.1 ± 0.8 for the subthreshold depression group, with no significant differences observed among the three groups **Table 2**.

**Table 2:** Clinical characteristics of patients.

	MDD (n=51)	BD (n=35)	Subthreshold depression (n=36)	P-value
Duration of illness, years, mean (SD)	10.1 (7.7)	10.3 (7.9)	11.4 (7.8)	0.61
Psychiatric Comorbidities				
Anxiety disorders, n (%)	17 (33.3%)	12 (34.3%)	16 (44.4%)	0.53
Somatoform disorders, n (%)	1 (2.0%)	1 (2.9%)	6 (16.7%)	<0.01
Asperger's disorder, n (%)	5 (9.8%)	2 (5.7%)	9 (25.0%)	0.04
ADHD, n (%)	8 (15.7%)	6 (17.1%)	10 (27.8%)	0.34
Alcohol use disorders, n (%)	2 (3.9%)	5 (14.3%)	1 (2.8%)	<0.01
Eating disorders, n (%)	4 (7.8%)	3 (8.6%)	0	0.21
Personality disorders, n (%)	39 (76.5%)	27 (77.1%)	18 (50.0%)	0.01
Paranoid	9 (17.6%)	6 (17.1%)	1 (2.8%)	0.09
Schizotypal	4 (7.8%)	6 (17.1%)	0	0.03
Schizoid	1 (2.0%)	1 (2.9%)	5 (13.9%)	0.04
Borderline	16 (31.4%)	14 (40.0%)	4 (11.1%)	0.02
Narcissistic	7 (13.7%)	8 (22.9%)	4 (11.1%)	0.35
Antisocial	4 (7.8%)	2 (5.7%)	0	0.24
Dependent	8 (15.7%)	3 (8.6%)	1 (2.8%)	0.13
Obsessive-compulsive	22 (43.1%)	19 (54.3%)	5 (13.9%)	<0.01
Avoidant	17 (33.3%)	13 (37.1%)	3 (8.3%)	<0.01
Passive-aggressive	6 (11.8%)	7 (20.0%)	4 (11.1%)	0.47
Depressive	24 (47.1%)	9 (25.7%)	7 (19.4%)	0.01
Mental retardation, n (%)	0	0	2 (5.6%)	0.09
Schizophrenia, n (%)	0	0	2 (5.6%)	0.09
MADRS, mean (SD)	20.9 (9.7)	18.6 (9.3)	11.3 (7.4)	<0.01
YMRS, mean (SD)	1.7 (3.0)	4.4 (6.3)	2.2 (2.9)	0.08
ASRS, mean (SD)	2.8 (1.5)	2.7 (1.6)	2.5 (1.9)	0.55
AQ, mean (SD)	23.7 (7.2)	23.3 (8.4)	21.8 (7.7)	0.52
WAIS-III				
VIQ, mean (SD)	105.1 (13.6)	101.4 (16.2)	103.9 (14.7)	0.37
PIQ, mean (SD)	100.1 (13.2)	96.4 (13.2)	96.1 (17.9)	0.22
FIQ, mean (SD)	103.2 (13.1)	99.2 (15.4)	100.4 (16.7)	0.26
The number of past antidepressant treatment failure, mean (SD)	1.20 (0.92)	0.86 (0.88)	1.11 (0.79)	0.20

ADHD, attention-deficit hyperactivity disorder; AQ, Autism Spectrum Quotient; ASRS, Adult ADHD Self-Report Scale; BD, bipolar disorder; FIQ, full intelligence quotient; MADRS, Montgomery-Asberg Depression Scale; MDD, major depressive disorder; PIQ, performance intelligence quotient; SD, standard deviation; VIQ, verbal intelligence quotient; WAIS-III, Wechsler Adult Intelligence Scale, Third Edition; YMRS, Young Mania Rating Scale.

## 4 Discussion　

This study is the first to investigate the sociodemographic and clinical characteristics of patients with suspected DTD, including developmental traits and intelligence quotient (IQ), in real-world settings. In our study, 40% of the patients were diagnosed with MDD according to the SCID, 30% with BD, and the remaining 30% with subthreshold depression, usually presenting with a combination of various psychiatric disorders. No significant differences were observed in sociodemographic and clinical factors among these three groups except for the comorbidity of somatoform disorders, alcohol use disorders, and some personality disorders along with the MADRS scores. These results underscore the importance of comprehensive assessments in the management of suspected DTD, attributing to the multifaceted nature of this condition, to facilitate the provision of tailored treatment strategies.

In this study, we observed that approximately 30% of patients with suspected DTD were diagnosed with BD, while MDD accounted for 40%. These findings indicate that BD as well as MDD is prevalent primary diagnoses in suspected DTD. It is well-documented that BD is characterized not only by alternating episodes of depression and mania but also by prolonged depressive phases (14). Moreover, distinguishing BD from MDD poses a clinical challenge due to overlapping demographic characteristics, such as age and gender (15). Notably, prior research indicates that between 37% and 69% of individuals eventually diagnosed with BD were initially diagnosed with unipolar depression, and it takes approximately 1.5 to 7 years for a BD diagnosis to be confirmed (16–19). While certain studies have highlighted clinical, biochemical, imaging, and genetic differences between these disorders, no single factor has been consistently definitive in routine clinical practice (15). The variability in IQ between MDD and BD remains a subject of debate. While certain cross-sectional studies indicate that individuals with BD may have higher IQs than those with MDD (20), contrasting findings have emerged from another research. For instance, one study involving 1728 hospitalized patients and another encompassing 450 patients revealed either no marked difference in IQ between BD and MDD or lower IQ scores in BD patients across a range of subtests and full-scale IQ (21, 22). Our study did not find significant sociodemographic or clinical characteristics to distinctly differentiate MDD, BD, and subthreshold depression in suspected DTD patients, except for some psychiatric comorbidities and the MADRS scores. This underscores the diagnostic complexity of distinguishing between MDD and BD in suspected DTD cases. Considering the different treatment protocols recommended for these conditions, our results highlight the critical need for meticulous and accurate clinical evaluations.

Thirty percent of the present suspected DTD patients were found to have subthreshold depression, with 95% of these individuals having some psychiatric disorder, including personality disorder, autism spectrum disorder, or ADHD. A cross-sectional study of 3,400 outpatient psychiatric patients, conducted using semi-structured interviews based on DSM-IV criteria, found that social anxiety and personality disorders were the most common comorbidities among 300 patients diagnosed with subthreshold depression (23). Furthermore, it is noteworthy that subthreshold depression can lead to adverse clinical outcomes. For example, a systematic review of 27 studies with adolescent subthreshold depression revealed that this condition is associated with significant functional impairment and reduced quality of life (24). In one observational study of 120 patients with subthreshold depression, 12% progressed to major depression over a three-year period (25). Another observational study, examining 320 patients hospitalized for suicide attempts, reported severe suicide attempts in 15% of those with subthreshold depression (26). Given the potential risks and chronic nature of depressive symptoms, close monitoring of patients diagnosed with subthreshold depression within the suspected DTD spectrum is essential.

Specifically, while significant differences were observed among the three groups in the prevalence of some personality disorders, the overall comorbidity of personality disorders was high, ranging from 50-77% for MDD, BD, and subthreshold depression. It has been proposed that the presence of comorbid personality disorders may contribute to a lower likelihood of remission and a higher likelihood of recurrence of depressive symptoms (27). The findings of the present study also suggest that comorbidity of personality disorders may be a contributing factor to the refractoriness of depressive symptoms.

There are several limitations that warrant caution in interpreting the findings of this report. First, the study population comprised exclusively of outpatients from a single university hospital in Japan. This specific demographic may limit the generalizability of the results to other clinical settings. Second, the limited sample size and the absence of a power calculation pose challenges for statistical power and the ability to detect smaller effect sizes. This limitation may affect the reliability and generalizability of the findings. Third, the data, collected between 2014 and 2018, may not fully represent the current trends in the manifestation of suspected DTD. Notably, the study relied on the DSM-IV-TR for diagnostic criteria, which may not encapsulate recent updates in understanding of psychiatric disorders as outlined in the DSM-5. Fourth, no assessment scales were utilized to measure the patients’ subjective burden in this study. Variations in patients’ awareness of their burden could potentially influence the objective evaluation data collected. Fifth, the study included patients initially treated for depression who were later diagnosed with BD. Although antipsychotics and mood stabilizers are the cornerstone of pharmacotherapy for BD, as outlined in various practice guidelines, this study did not record the patients’ histories of antipsychotic and mood stabilizer use, and therefore, such data cannot be presented. Finally, the cross-sectional nature of the study restricts our ability to infer causality or track the progression and treatment response over time. A longitudinal approach would offer deeper insights into the dynamics and trajectory of suspected DTD.

In conclusion, our investigation into suspected DTD reveals the complexity and variability in its clinical presentation, particularly highlighting the prevalence of MDD, BD, and subthreshold depression, in addition to several psychiatric comorbidities. Among the three groups, significant differences were not observed in sociodemographic or clinical characteristics, with the exception of certain psychiatric comorbidities and the MADRS scores. These findings emphasize the need for comprehensive evaluations and suggest the necessity for more extensive and longitudinal research to enhance our understanding and management of suspected DTD.

## Data Availability

The raw data supporting the conclusions of this article will be made available by the authors, without undue reservation.
